# RNAStat: An Integrated Tool for Statistical Analysis of RNA 3D Structures

**DOI:** 10.3389/fbinf.2021.809082

**Published:** 2022-01-11

**Authors:** Zhi-Hao Guo, Li Yuan, Ya-Lan Tan, Ben-Gong Zhang, Ya-Zhou Shi

**Affiliations:** ^1^ Research Center of Nonlinear Science, School of Mathematical and Physical Sciences, Wuhan Textile University, Wuhan, China; ^2^ School of Computer Science and Artificial Intelligence, Wuhan Textile University, Wuhan, China

**Keywords:** RNA 3D structure, statistical analysis, secondary structure motifs, non-canonical base pair, structure evaluation

## Abstract

The 3D architectures of RNAs are essential for understanding their cellular functions. While an accurate scoring function based on the statistics of known RNA structures is a key component for successful RNA structure prediction or evaluation, there are few tools or web servers that can be directly used to make comprehensive statistical analysis for RNA 3D structures. In this work, we developed RNAStat, an integrated tool for making statistics on RNA 3D structures. For given RNA structures, RNAStat automatically calculates RNA structural properties such as size and shape, and shows their distributions. Based on the RNA structure annotation from DSSR, RNAStat provides statistical information of RNA secondary structure motifs including canonical/non-canonical base pairs, stems, and various loops. In particular, the geometry of base-pairing/stacking can be calculated in RNAStat by constructing a local coordinate system for each base. In addition, RNAStat also supplies the distribution of distance between any atoms to the users to help build distance-based RNA statistical potentials. To test the usability of the tool, we established a non-redundant RNA 3D structure dataset, and based on the dataset, we made a comprehensive statistical analysis on RNA structures, which could have the guiding significance for RNA structure modeling. The python code of RNAStat, the dataset used in this work, and corresponding statistical data files are freely available at GitHub (https://github.com/RNA-folding-lab/RNAStat).

## 1 Introduction

RNA molecules play important roles in various biological processes, ranging from carrying genetic information, participating in protein synthesis, catalyzing biochemical reactions, and regulating gene expressions, to acting as a structural molecule in cellular organelles (Doherty and Doudna, 2001; Dethoff et al., 2012; Cech and Steitz, 2014). Generally, to perform functions, RNAs need to form special tertiary structures, which typically can be determined by experimental methods such as cryo-electron microscopy, X-ray crystallography, and nuclear magnetic resonance spectroscopy (NMR) (Fernandez-Leiro and Scheres, 2016; Rose et al., 2017; Westhof and Leontis, 2021). However, the structures deposited in Protein Data Bank (PDB) are still limited, since it is expensive and time-consuming to experimentally derive high-resolution RNA 3D structures (Rose et al., 2017; Westhof and Leontis, 2021). This situation has led to a great demand in structural biology to envisage the RNA structures using prediction methods (Hajdin et al., 2010; Shi Y.-Z. et al., 2014; Miao et al., 2017; Schlick and Pyle, 2017).

In the last decade, there are some computational models have been developed for predicting RNA 3D structures, among which the knowledge-based fragment assembly methods (Gan et al., 2004; Das and Baker, 2007; Parisien and Major, 2008; Das et al., 2010; Flores et al., 2010; Cao and Chen, 2011; Rother et al., 2011; Popenda et al., 2012; Zhao et al., 2012; Jian et al., 2019; Zhang et al., 2021) and the physics-based coarse-grained (CG) models have gained more attention (Jonikas et al., 2009; Flores and Altman, 2010; Pasquali and Derreumaux, 2010; Flores et al., 2012; Denesyuk and Thirumalai, 2013; Xia et al., 2013; Shi YZ. et al., 2014; Šulc et al., 2014; Krokhotin et al., 2015; Boniecki et al., 2016). For example, the FARNA/FARFAR can assemble trinucleotide fragments into 3D structures corresponding to an RNA sequence with the use of the Monte Carlo algorithm and a knowledge-based energy function, and the parameters of energy function were determined from the statistical analysis of known RNA 3D structures (Das and Baker, 2007; Das et al., 2010). The SimRNA with a CG representation, which employs a statistical potential derived from PDB structures, and can fold RNAs using only sequence information (Boniecki et al., 2016). Recently, we have also provided a new CG model to predict 3D structures and stability of an RNA in ion solutions from sequence alone (Shi Y.-Z. et al., 2014, 2015, 2018; Jin et al., 2019). Although the potential energy of our model is mainly physics-based, the potentials, especially bonded potentials, were also parameterized by the statistical analysis on the available 3D structures of RNAs in PDB (Shi YZ. et al., 2014; Jin et al., 2019).

Furthermore, the existing knowledge-based methods usually produce an ensemble of candidate structures, which should be further evaluated to recognize the best candidates as close to native structures as possible (Huang and Zou, 2011; Miao and Westhof, 2017; Yan et al., 2018; Tan et al., 2019; Magnus et al., 2020). To address this issue, several statistical potentials have been developed to evaluate RNA 3D structures (Bernauer et al., 2011; Capriotti et al., 2011; Wang et al., 2015; Li et al., 2016; Li et al., 2018; Masso, 2018; Yu et al., 2019; Zhang et al., 2020), such as RASP (Capriotti et al., 2011), RNA KB potentials (Bernauer et al., 2011), 3dRNAscore (Wang et al., 2015), and DFIRE (Zhang et al., 2020). Generally, these potentials are proportional to the frequencies of occurrence of atom pairs, angles, or dihedral angles in PDB structures based on Boltzmann or Bayesian formulations (Huang and Zou, 2011; Yan et al., 2018; Tan et al., 2019). For example, Capriotti et al. have built the RASP by calculating the density distribution of distance between any two atoms in all the known RNA structures (Capriotti et al., 2011). The 3dRNAscore introduced by Wang et al. uses seven typical RNA dihedral angles as well as distance-dependent geometrical descriptions for atom pairs to construct the statistical potentials (Wang et al., 2015). In addition to structure evaluation, very recently, Xiong et al. have proposed a fully knowledge-based function (BRiQ) based on statistics of orientation distribution of one base around another base from the PDB structures for improving RNA model refinement (Xiong et al., 2021).

Obviously, all these advances on RNA structure modeling indicate that to gather various statistics of RNA 3D structures is generally essential to predict RNA tertiary structures. However, there are few tools or web servers that can be used to make comprehensive statistical analysis for RNA 3D structures (Andronescu et al., 2008; Cock et al., 2009; Baulin et al., 2016; Danaee et al., 2018; Magnus et al., 2020). Recently, Baulin et al. have proposed a database URSDB (the Universe of RNA structures database) to store information (e.g., annotations of main structural elements) obtained all RNA-containing PDBs (Baulin et al., 2016). Although the URSDB can allow the user to get statistics on structural motifs (base pairs, stems, and loops) based on the information provided by the software of DSSR (dissecting the spatial structure of RNA) (Lu et al., 2015; Lu, 2020), these statistics on RNA secondary structure motifs could be far from enough to help RNA 3D structure modeling (Miao and Westhof, 2017; Tan et al., 2019). Fortunately, several works have provided statistics of RNA structures from different aspects. For example, both the RNA 3D Motif Atlas and bpRNA can provide a statistical summary of the hairpin and internal loop motifs (Parlea et al., 2016; Danaee et al., 2018). The RNA STRAND can also provide information on structural features such as types and sizes for stems and loops (Andronescu et al., 2008). To build scoring function for RNA structure prediction, Bottaro et al. as well as Das and Baker have developed methods to calculate the geometrical properties of RNA base-pairing and base-stacking (Bottaro et al., 2014; Das and Baker, 2007). Despite all this progress, with the rapidly increasing number of RNA structures deposited in PDB (Supplementary Figure S1 in the Supplementary Material) (Rose et al., 2017; Westhof and Leontis, 2021), an available tool to convenient access comprehensive statistical information of RNA 3D structures is still necessary.

Here, we present a novel tool, named as RNAStat, special for the statistical analysis of RNA 3D structures. It can be used to calculate structural information of RNA 3D structure(s) at different levels: global 3D structural level, secondary structure level, and atomic level. We first introduced the function and principle of the RNAStat. Afterward, based on a non-redundant RNA structure dataset established by us, we utilized the RNAStat to perform statistical analysis for RNA 3D structures, and provided various statistical data of RNA structural properties (e.g., size/shape, geometry of base-pairing/stacking, secondary structure motifs, and atom-atom distance). Throughout the article, we also discussed the potential value of these statistics on RNA 3D structure prediction and evaluation.

## 2 Materials and Methods

**FIGURE 1:**
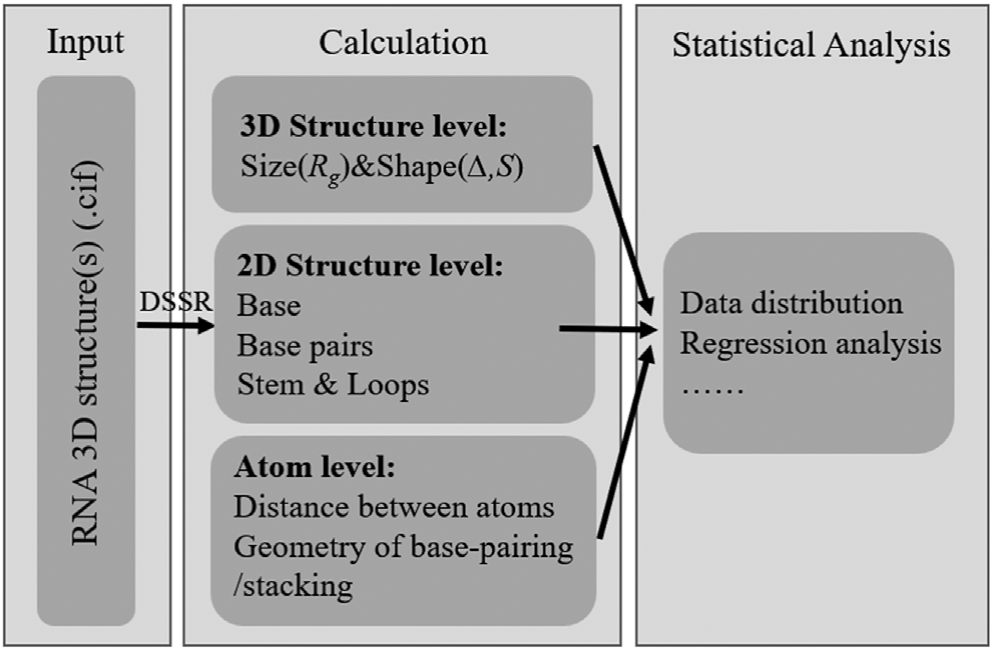
The basic functions of RNAStat for RNA 3D structure calculation and statistical analysis.

The RNAStat provided in this work can be used to make calculation (or statistics) for given RNA structure(s) in the following aspects: 1) the radius of gyration (i.e., size): and shape; 2) the secondary structure motifs; 3) the geometry of base-pairing and base-stacking; 4) the distances between atoms; see Figure 1.

### 2.1 Radius of Gyration

The mean radius of gyration $R_{g}$ is often used as geometric measure of the size of RNA as well as DNA and protein (Hyeon et al., 2006; Rawat and Biswas, 2009), since it can be easily determined by experimental methods such as small angle neutron scattering or X-ray scattering. For RNAs, it is possible to assume equal masses for all nonhydrogen atoms, so that the $R_{g}^{2}$ of a given RNA 3D structure (in PDB format, e.g.,.cif) can be calculated by (Hyeon et al., 2006)

$$\langle R_{g}^{2}\rangle =\frac{1}{N}\sum_{i=1}^{N}\langle {\left(r_{i}-r_{0}\right)}^{2}\rangle$$ (1)

where *N* is the number of heavy atoms (C, P, N, and O) in the RNA molecule, $r_{i}$ is the position of the *i*th atom. The $r_{0}$ in Eq. 1 represents the coordinates of the geometric center of RNA, calculated using $r_{0}=\frac{1}{N}\sum_{i=1}^{N}r_{i}$ .

### 2.2 Shape

**FIGURE 2:**
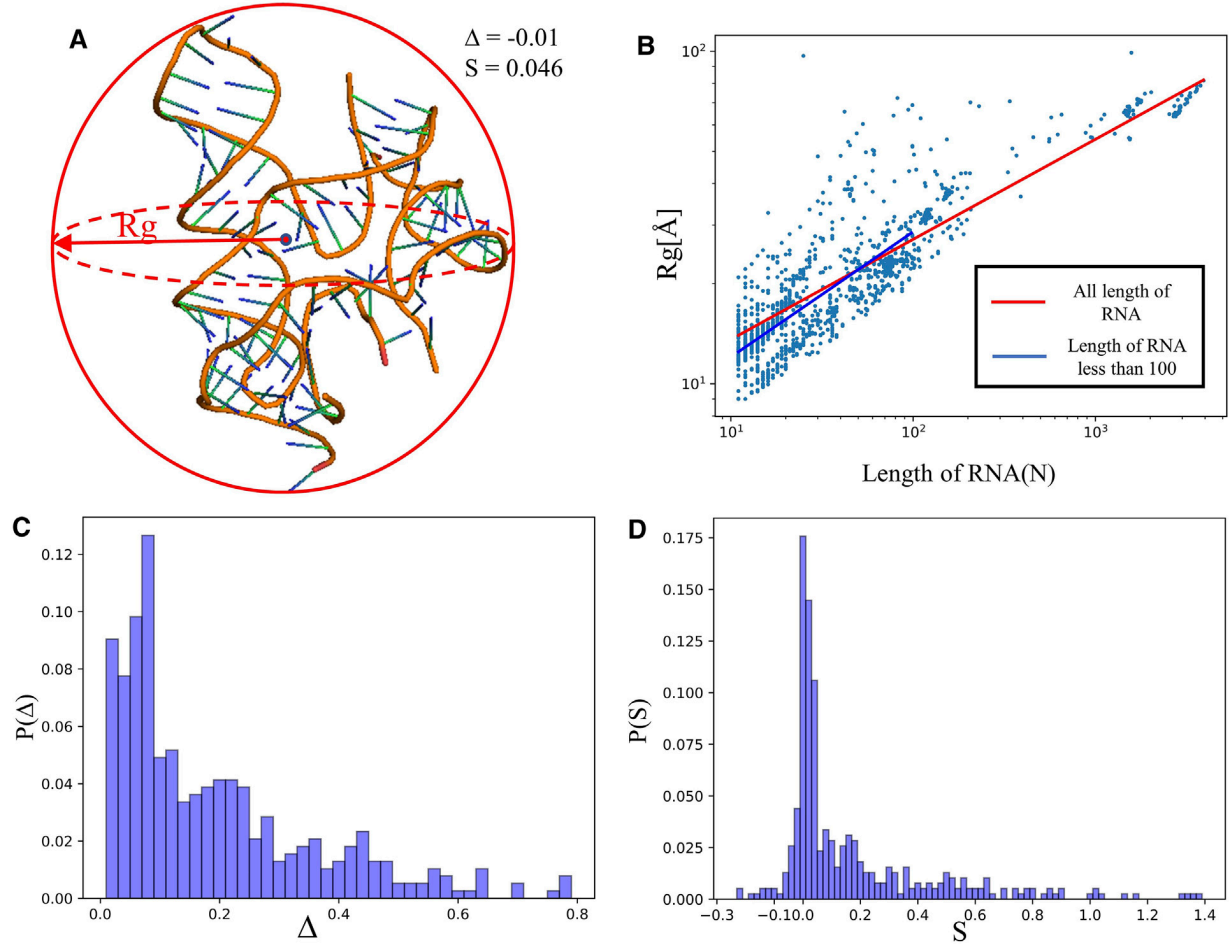
**(A)** The schematic diagram of size and shape for an RNA 3D structure (PDB ID: 4QLM). $R_{g}$ is the radius of gyration, Δ represents the asphericity parameter and S is the shape parameter, and their values are calculated by the RNAStat (Eqs 1–4). The 3D structure of 4QLM is shown with the PyMol (http://www.pymol.org). **(B)** Radius of gyration ( $R_{g}$ ) as a function of length of RNA (*N*). Dots: $R_{g}^{'}s$ of RNA structures in our dataset. Red line: the best-fit line to the data that shows the scaling law $R_{g}=6.7L^{0.31}$ . Blue line: the best-fit line ( $R_{g}=5.1L^{0.37}$ ) to the data for RNAs with length less than 100 nt. **(C, D)** Distributions of the asphericity parameter Δ **(C)** and shape parameter S **(D)** for RNA structures in our dataset.

Since the shape of RNAs is rather important in determining the overall motion of RNA and their interaction with other biomolecules, two rotationally invariant quantities, the asphericity parameter Δ and shape parameter S , and are used to characterize the deviation of an RNA conformation from the spherical shape (Figure 2A) (Hyeon et al., 2006). Based on the Refs. (Hyeon et al., 2006; Rawat and Biswas, 2009), the Δ and S can be determined from the inertia tensor,

$$T_{\alpha \beta }=\frac{1}{2}\sum_{i=1}^{N}\sum_{j=1}^{N}\left(r_{i\alpha }-r_{j\alpha }\right)\left(r_{i\beta }-r_{j\beta }\right)$$ (2)

where α, β=x, y, z , are the coordinate component, and $r_{i\alpha }$ is the α -th component of the position of the *i*th atom. Due to the $R_{g}^{2}=trT$ , the eigenvalues $\left(\lambda_{1},\lambda_{2},\lambda_{3}\right)$ of the matrix $T_{\alpha \beta }$ are the squares of the three principal radii of gyration. Thus, the Δ and S can be directly calculated by

$$S=\frac{27\prod_{i=1}^{3}\left(\lambda_{i}-\lambda \right)}{{\left(trT\right)}^{3}}$$ (3)

$$\Delta =\frac{3}{2}\frac{\sum_{i=1}^{3}{\left(\lambda_{i}-\lambda \right)}^{2}}{{\left(trT\right)}^{2}}$$ (4)

where $\lambda =\left(\lambda_{1}+\lambda_{2}+\lambda_{3}\right)/3$ . As shown in Eqs 2–4, the shape parameter S measures the prolateness or oblateness of a conformation and the asphericity parameter Δ characterizes the average deviation of the conformation from spherical symmetry. The S satisfies the bound −1/4≤S≤2 , and S>0 represents prolate ellipsoid, S<0 corresponds to oblate ellipsoid, while S=0 infers symmetric sphere. The Δ is in the range of [0, 1], where Δ=0  means that the RNA molecule is a perfect sphere, and otherwise, the value of Δ indicates the extent of anisotropy.

### 2.3 Secondary Structure Motifs

**FIGURE 3:**
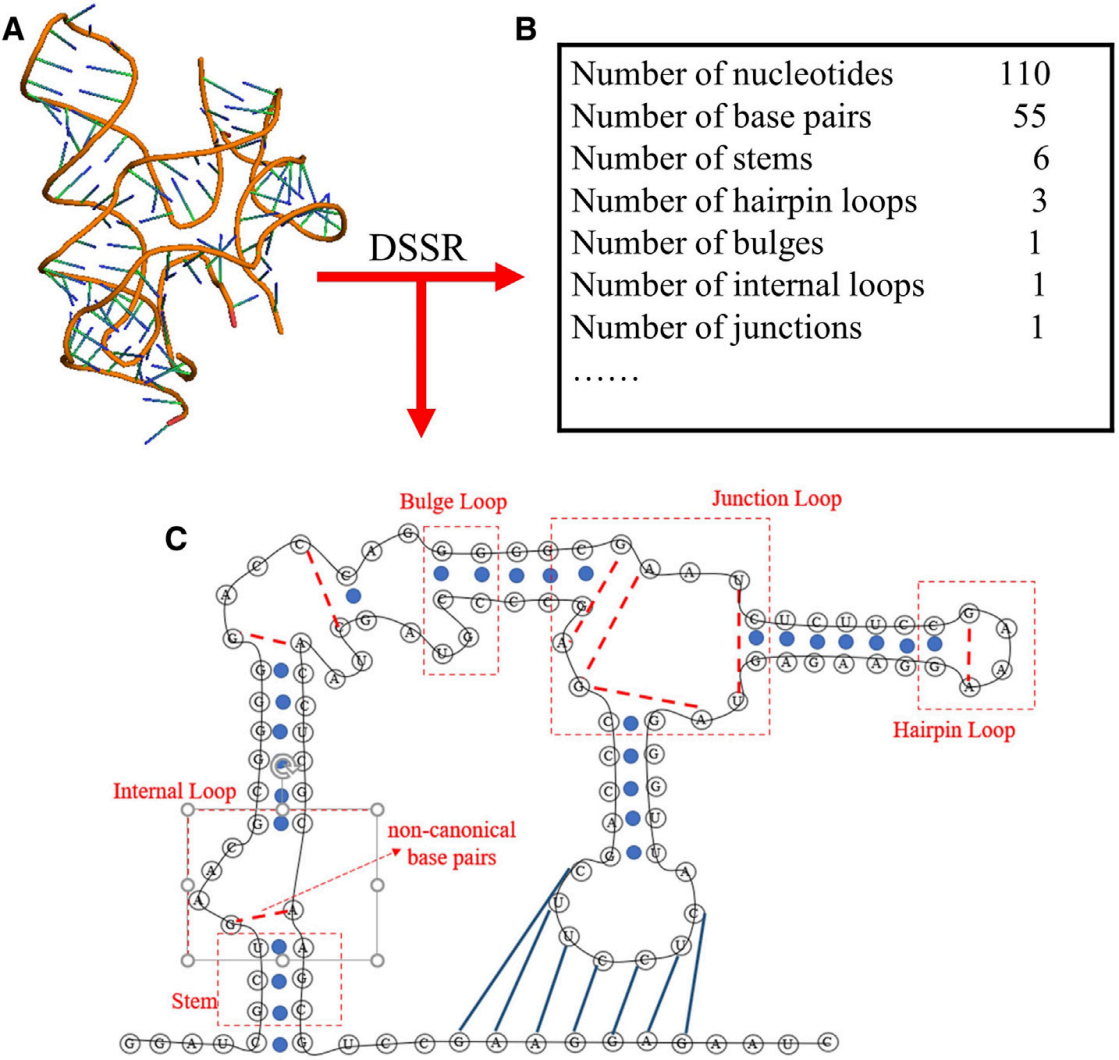
The schematic diagram of the secondary structure information extracted from an RNA 3D structure (ydaO riboswitch, PDB ID: 4QLM) using DSSR software in RNAStat. **(A)** 3D structure shown with the PyMol (http://www.pymol.org) for the RNA. **(B)** The DSSR software is called to analyze the RNA 3D structures, e.g., for the RNA, the secondary structure information including the details of canonical/noncanonical base pairs, stems, and various loops. **(C)** The secondary structure drawn based on the secondary structure information from **(B)** for the RNA. Black lines: backbone. Blue solid circles and blue solid lines: canonical base pairs (A-U, G-C, and G-U). Red dotted lines: non-canonical base pairs. Dashed boxes: samples of secondary structural motifs.

To obtain the secondary structure motifs for an RNA PDB structure, the RNAStat can directly call the DSSR through the corresponding python command (e.g., x3dna-dssr.exe--json “+ ”-o = file); The DSSR is an integrated and automated command-line tool for analysis and annotation of RNA tertiary structures, and it can characterize nucleotides, base pairs, pseudoknots, loops, stems, and coaxially stacked helices (Lu et al., 2015; Lu, 2020); see an example in Figure 3. Based on the information extracted from DSSR, for an RNA structure set, the RNAStat can further provide the statistics of secondary structural elements, including base-pairs, stems, and various loops. In this work, we considered all C-G, A-U and G-U pairs to be canonical base pairs, and all other base pairs to be non-canonical ones, and the definitions of the secondary structural motifs can be found everywhere (Leontis and Westhof, 2001) and the simple illustration of them are also shown in Figure 3.

### 2.4 Geometry of Base-Pairing and Base-Stacking

**FIGURE 4:**
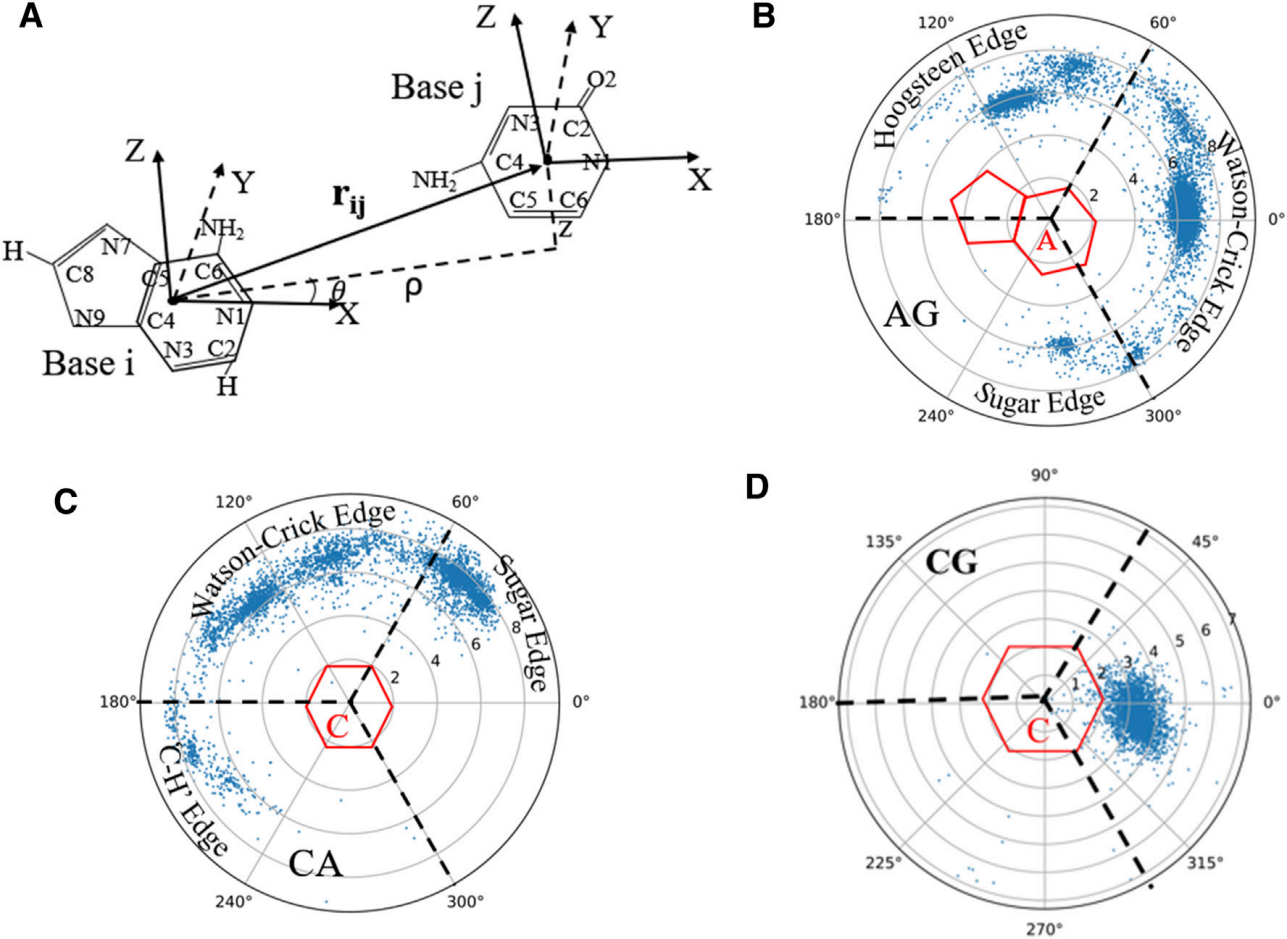
**(A)** The definition of the local coordinate system for bases, and in the coordinate system of one base (e.g., *i*), the position of another base (e.g., *j*) can be described by the vectors $r_{ij}$ , expressed in cylindrical coordinates. **(B)** Distribution of (ρ,θ) for base U near its paired base A. **(C)** Distribution of (ρ,θ) for base A near its paired base C. **(D)** Distribution of (ρ,θ) for the base G near its stacked base C. In **(B–D)**, the three interacting edges of each base (Watson–Crick, Hoogsteen/C-H, and sugar) correspond to positions in the three sectors of the map demarcated by the dotted lines, and the dots are from the statistics on the RNA structures in our dataset (see in Materials and Methods).

Since base-pairing and base-stacking are critical interactions that stabilize RNA 3D structures (Butcher and Pyle, 2011; Bottaro et al., 2014; Wang et al., 2016; Wang et al., 2020), the RNAStat can calculate the geometry between two bases in base-pairing/stacking. First, the whole nucleobase (i.e., A, U, G, and C) is treated as a single rigid group, and a coordinate system is set up on each base, with the origin (O) at the geometric center of all the heavy atoms. Similar to the local referential of a nucleotide introduced by Gendron et al. (2001), for pyrimidines (or purines), the two unit vectors, u between coordinates of atom N1 and C8 (C4 in purines), and v between coordinates of atom N1 and N3, can be built, and the unit vector Z is oriented along the cross product u×v . The unit vector X is built between coordinates of the origin (O) and atom N1, and the unit vector Y is given by Z×X ; see Figure 4A. Following this definition, the position of base *j* in the coordinate system constructed on base *i* is described by the vector ***r***_***ij***_, which can be conveniently expressed in cylindrical coordinates (*ρ,θ,z*) (Gendron et al., 2001; Das and Baker, 2007; Flores et al., 2011; Bottaro et al., 2014). And then, the geometry of pairing and stacking bases can be described by the distance *ρ* and angle *θ*. Based on the information of base-pairing from DSSR, the distributions of *ρ* and *θ* can be used to characterize the geometry of different base pairs including canonical and non-canonical Watson-Crick base pairs as well as those interacting through the Hoogsteen or sugar edge; see Figures 4B,C. The definitions of different types of base-pairing can be found in Ref. (Leontis and Westhof, 2001). and Supplementary Figure S2 in the Supplementary Material. Similarly, the stacking geometric property between two neighboring bases can also be characterized by *ρ-θ* planes (Figure 4D).

### 2.5 Distance Between Any Two Atoms

As described in the Introduction, the most existing statistical potentials for RNA structure evaluation are based on the distances between various type atoms (Miao and Westhof, 2017; Tan et al., 2019). Based on the coordinates of all the heavy atoms in an RNA structure (i.e.,.cif file), the distance $d_{ij}^{ab}$ between any two atoms *i* and *j* with types of *a* and *b*, respectively, can be simply calculated in Cartesian coordinate by:

$$d_{ij}^{ab}=\sqrt{{\left(r_{ai}-r_{bj}\right)}^{2}}$$ (5)

where $r_{ai}\;$ is the coordinates of the *i*th atom with type of *a* (e.g., P and C4′). In the RNAStat, there are two modes for users to choose: 1) calculating distances between atoms specified by the user; 2) calculating all distances between any two types of atoms. In addition to the calculation of distance, the RNAStat can automatically output the distribution of the distance between two atom types, and which could be directly used to construct distance-based statistical potential (Capriotti et al., 2011; Wang et al., 2015; Tan et al., 2019).

### 2.6 Dataset Used in This Work

To test the RNAStat, we established a non-redundant dataset based on the RNA 3D Hub set (Release nrlist_3.157_4.0 Å), in which the sequence identity between any two chains in the set is less than 95% (Leontis and Zirbel, 2012). Firstly, we collected 1,245 representative RNAs of all the different clusters with a resolution <4.0 Å from RNA 3D Hub list, which can be downloaded from http://rna.bgsu.edu/rna3dhub/nrlist. Then, we deleted the structure of non-RNA strands in the dataset. Afterwards, we removed the RNA structures with sequence identity ˃ 80% using the BLASTN program (Camacho et al., 2009). Finally, through the prior operation steps, 748 RNA structures were retained and their 3D structure files were downloaded from the PDB. The final RNA structure dataset used in this work can be found in the Supplementary Material as well as at GitHub (https://github.com/RNA-folding-lab/RNAStat), including PDB IDs, and PDB CIF files.

## 3 Results and Discussion

### 3.1 Overview of the RNAStat

In this work, we present the RNAStat, an integrated tool for making comprehensive statistics on RNA 3D structures. As shown in Figure 1, the RNAStat can be used to do statistical analysis for RNA 3D structures at different levels, such as global 3D structure level, secondary structure level, and atom level. The code of the RNAStat in python can be found at GitHub through https://github.com/RNA-folding-lab/RNAStat. In the following, we will give a brief introduction of the usage method of the tool.

The input to RNAStat is the coordinate file(s) of RNA 3D structure(s) in CIF format. Based on the needs of users, the input can be a single PDB file of an RNA structure or the PDB files for a given RNA structure set. For each PDB file, the RNAStat can calculate the size and shape of the RNA through Eqs 1–4 (in section of Materials and Methods), and call the DSSR to obtain its secondary structure motifs, e.g., the information of base-pairs, stems and various loops; see Figure 3. In the RNAStat, the distance between any heavy atom pair can also be calculated by Eq. 5, and the atom pair types can be specified by the user or default to all kinds of atom types, where 85 heavy atom types in four nucleotides (A, U, G, and C) are considered (Wang et al., 2015; Tan et al., 2019); see Supplementary Table S2 in the Supplementary Material. In addition, based on the information of base-pairing and the coordinates of atoms in two paired bases, the geometrical properties of base-pairing and base-stacking can also be calculated.

More importantly, for RNA structure set, the RNAstat can provide statistical information for all the above structural properties as well as the frequency distribution of various base pairs, which could be directly used to build statistical potentials for RNA structure evaluation or refinement (Miao et al., 2017; Tan et al., 2019; Xiong et al., 2021). The details of the methods for the calculations and statistical analysis can be found in section of Materials and Methods.

### 3.2 Test on the RNA Structure Set

To show the applicability of the RNAStat tool, we established a non-redundant RNA 3D structure dataset (see Materials and Methods), and took it as an example for RNA 3D structure analysis and statistic. Simultaneously, based on the RNA structure set, we also provided various statistical results of RNA structures, and which could contribute to building RNA statistical potentials or energy function of RNA CG models.

#### 3.2.1 Size and Shape of RNA Structures

We calculated the radius of gyration $R_{g}$ for the 748 RNA structures in the dataset using Eq. 1, and found that $R_{g}$ generally increases with RNA length *L*; seen in Figure 2B. Further regression analysis showed that $R_{g}$ of RNA structures can be calculated by

$$R_{g}=6.7L^{0.31},$$ (6)

indicating that $R_{g}$ of folded RNA structures follows the Flory scaling law (Tanner, 2016; Hyeon et al., 2006). Although this is in accordance with the result from Hyeon et al. (i.e., $R_{g}=5.5L^{1/3}$ ) (Hyeon et al., 2006), the parameters are slightly different. The reasons may be that the RNA structures in our non-redundant dataset are more diverse, and each $R_{g}$ is calculated based on the entire RNA structure no matter how many chains in the RNA, instead of based on each RNA chain. As shown in Supplementary Figure S3 in the Supplementary Material, the length of most RNAs in dataset is in the range of (10, 100). The corresponding regression equation for these short RNAs is $R_{g}=5.1L^{0.37}$ (Figure 2B), suggesting that the length-dependence of structure size is relatively weak for long RNAs due to the more compact conformations. In addition, since RNA is a polyelectrolyte, its size also depends on the ion concentration (Woodson, 2005; Tan and Chen, 2006; Tan et al., 2015), which is one of the reasons why the $R_{g}^{'}s$ of RNAs with same length have a significant difference.

Figure 2C depicts the distribution of asphericity parameter ∆ of RNA structures in the dataset, where ∆ spans over the whole range from 0 to 0.8, and ∼60% has ∆<0.2, suggesting that RNAs are mostly spherical in nature (Hyeon et al., 2006; Tan et al., 2015). The distribution of the shape parameter S for RNA structures is displayed in Figure 2D. The plot exhibits that almost all RNAs have S > 0, and the distribution has a significant peak around S = 0, implying that RNAs do not deviate much from the spherical symmetry. Our statistics on ∆ and S are very close to the results from RNA complexes reported in Ref. (Hyeon et al., 2006), while are with the different from those of single-chain RNAs.

#### 3.2.2 Statistics on RNA Secondary Motifs

Since RNA structure formation is generally hierarchical (Brion and Westhof, 1997), the information of RNA secondary structures could be the key to evaluate or predict RNA tertiary structures. The DSSR software can be called by the RNAStat to analyze all the RNA tertiary structures in the dataset; see Figure 3. Based on the results from DSSR, various statistics on RNA secondary motifs can be showed.

**FIGURE 5:**
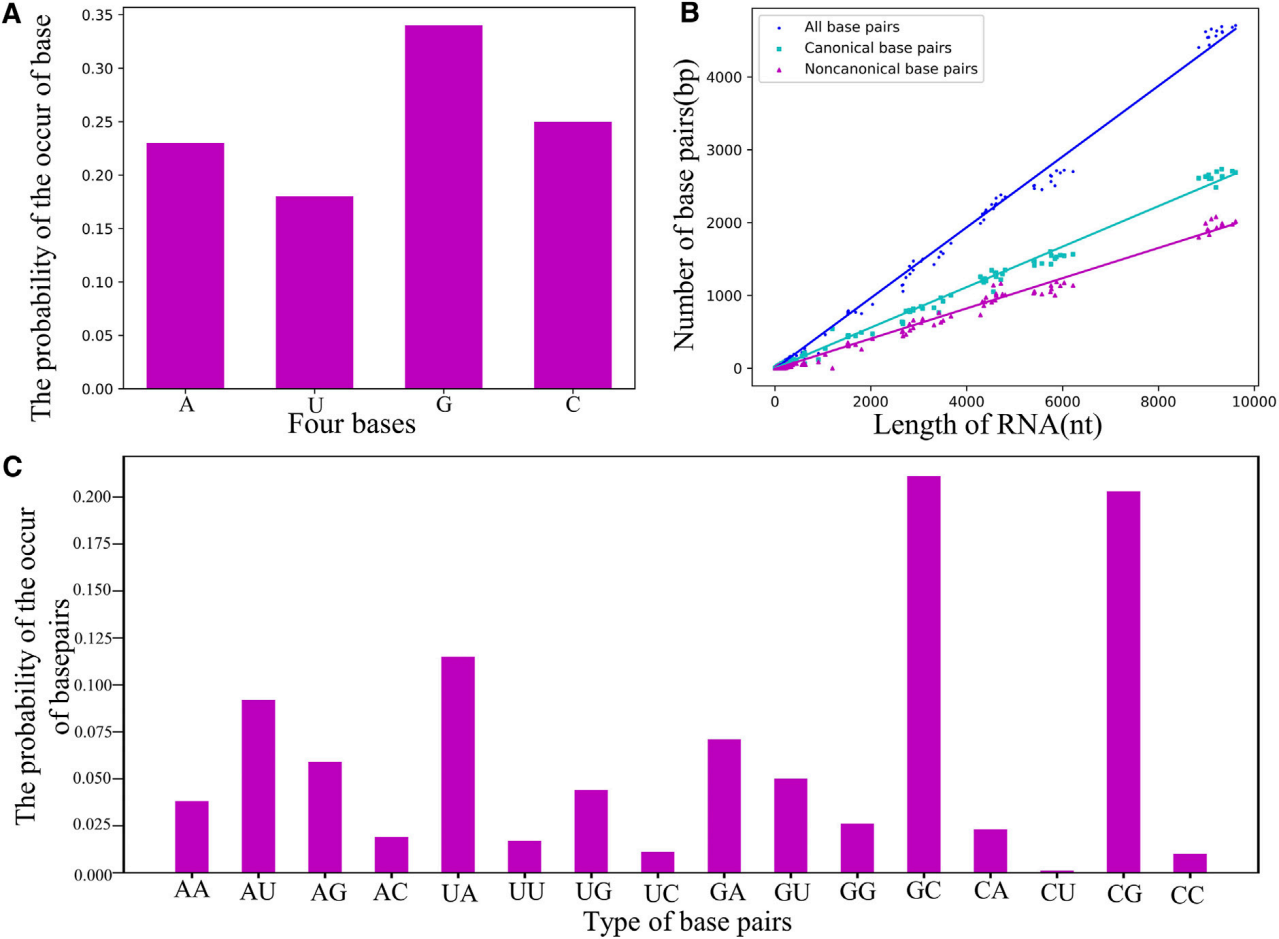
**(A)** The probability of the occurrence of nucleotides in the non-redundant dataset. **(B)** The counts of base-pairs as a function of length *N* for RNA structures in the dataset. Green squares: canonical base pairs. Purple triangle: non-canonical base pairs. Blue circle: all canonical and non-canonical base pairs. **(C)** The probability of the occurrence of base pairs including canonical and non-canonical ones.

As shown in Figure 5; Supplementary Tables S3–S5 in the Supplementary Material, the guanine nucleotide (i.e., G) and the base pairs of G-C/C-G are the most common in the RNA dataset, e.g., the probability of occurrence of G (∼34%) is apparently higher than that of the other bases. Using the dataset of RNA structures, we found that the number of base pairs $N_{bp}$ grows linearly with the sequence length L with the slope as ∼0.48 (i.e., $N_{bp}=0.48L$ ), and the number of non-canonical base pair $N_{bp}^{Non}$ also increases significantly with L: $N_{bp}^{Non}=0.21L$ ; see Figure 5B, suggesting that interaction of non-canonical base-pairing is rather important in 3D structure modeling for RNAs, especially for large RNAs (Das et al., 2010; Tan et al., 2015).

Figure 5C shows the probability of the occurrence of base pairs including canonical and non-canonical base pairs; seen also in Supplementary Table S4 in the Supplementary Material, and due to the proportional relation between base-pairing strength and their relative probability, this statistic of base pairs can be directly used to parameterize the base-pairing energy function for RNA models. For example, based on the relative probability between G-C/C-G (∼40%) and A-U/U-A (∼20%), we have set that the energy of G-C is twice the strength of the A-U in our CG model (Shi Y.-Z. et al., 2014; Jin et al., 2019), and the common non-canonical base pairs (e.g., A-G, A-A, and G-G) will be further taken into account. In addition, base-pair stacking make a significant contribution to the stability of an RNA structure (Schlick and Pyle, 2017; Miao and Westhof, 2017; Brion and Westhof, 1997; Laing and Schlick, 2009), and the stacking interaction parameters can also be obtained from the statistical frequency of base-pair stack (Supplementary Table S5 in the Supplementary Material), which could improve the predictions of RNA secondary (or 3D) structures and their thermodynamic stability (Dima et al., 2005; Gardner et al., 2011; Sloma and Mathews, 2017).

**FIGURE 6:**
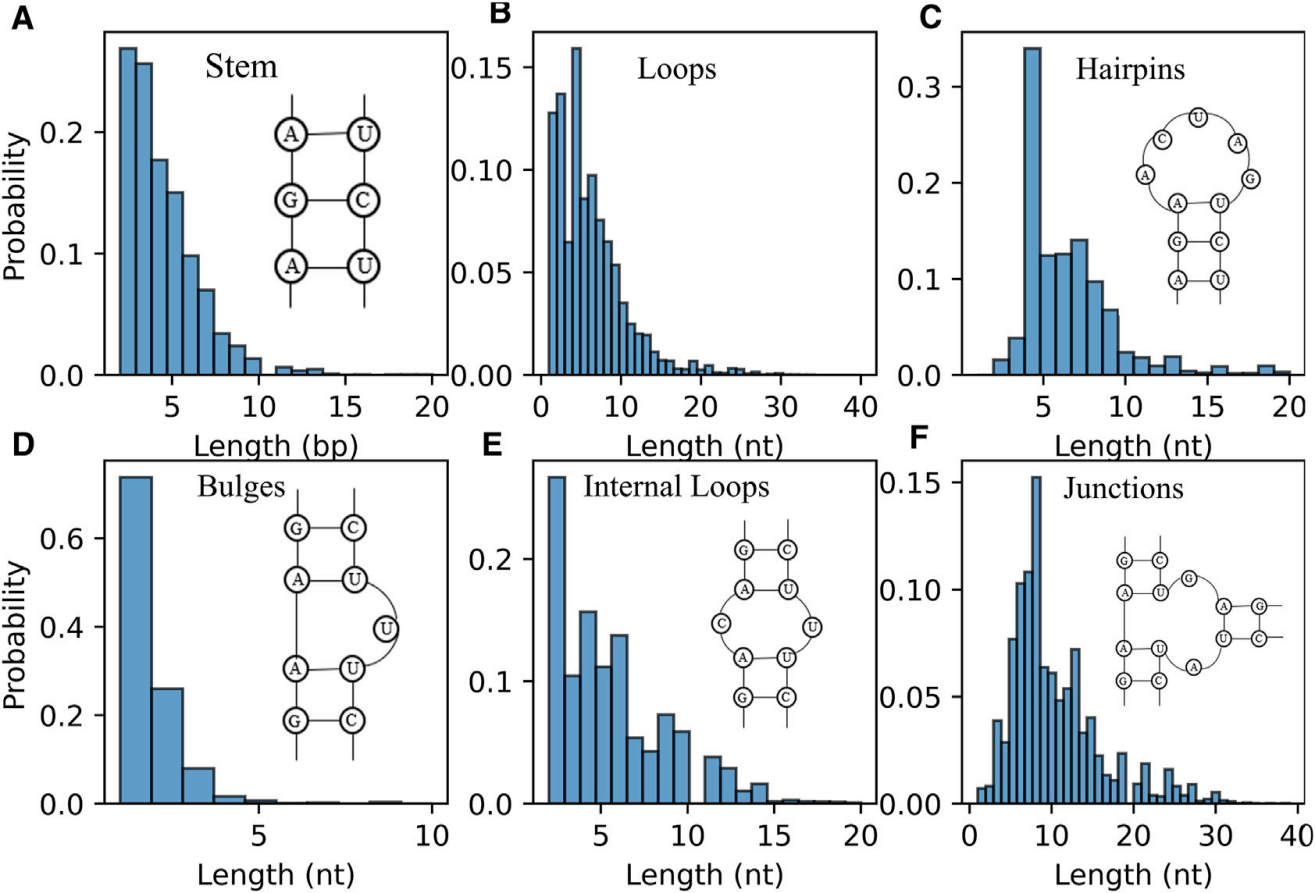
The distribution of length of RNA secondary structure motifs in the dataset. **(A)** Histogram of the occurrence for the length of stems. **(B–F)** Histogram of the occurrence for the length of loops **(B)** all loops; **(C)** hairpin loops; **(D)** bulge loops; **(E)** internal loops; **(F)** junction loops.

Furthermore, the distribution of length of RNA secondary structure motifs (e.g., stem and loops) could be helpful in the evaluation of structures predicted by *ab initio* models (Brion and Westhof, 1997; Danaee et al., 2018). Figure 6A displays the distribution of the length of stem, which is defined by the number of continuous canonical base pairs (Lu et al., 2015). Although the distribution of stem length for the RNAs in dataset is very broad, there is a prominent peak around ∼2 bp and the length of stem greater than 10 bp occur much less frequently; see Figure 6A, suggesting that stems are constantly interrupted by loops (Figure 6B) (Danaee et al., 2018). For hairpin loops shown in Figure 6C, we found that hairpin loops are most likely to have a length of 4 nt, i.e., tetraloops, which have been proved to be extremely stable by thermodynamic experiments (Butcher and Pyle, 2011), and the heptaloops (i.e., hairpin loops of length 7 nt) are the second most frequent, in line with the results from bpRNA, and RNA 3D Motif Atlas (Danaee et al., 2018; Parlea et al., 2016). On the contrary, the distribution of the bulge loop length only has one very significant peak at 1 nt, and almost all the bulge loops are with length less than 5 nt; seen in Figure 6D. The reasons could be that one stem interrupted by short bulge loops (e.g., 1 nt) is generally as stable as continuous helix with same sequence due to the coaxial-stacking interaction between two stems (Shi et al., 2015; Butcher and Pyle, 2011), while the stability of RNAs is reduced with the increase of the length of bulge loop (Zhang et al., 2019). As shown in Figures 6E,F, the distributions of internal/junction loop lengths are more complex, with more than one broad peak. For example, there are about four visible peaks observed for internal loop at 2, 4, 6, and 9 nt, respectively. Since the bases in two sides (5′ and 3′) of an internal loop often pairing together in non-canonical way, the internal loops often tend to be symmetric in order to keep a more stable structure (Laing and Schlick, 2009; Butcher and Pyle, 2011; Gardner et al., 2011). However, we only calculated the length of the entire loop without distinguishing 5′ and 3’ loop sequences, for simplicity in the present version of the RNAStat. More detailed statistics of internal/multi-loops should be taken into account in the future to help improve their energy parameters calculation.

#### 3.2.3 Statistics on Geometry of Base-Pairing and Base-Stacking

On account of the importance of the geometrical configuration of base-pair/stacking in RNA 3D modeling (Das and Baker, 2007; Bottaro et al., 2014), the RNAStat provides the calculation or statistic of geometry of base-pairing/stacking for RNA structures; see the section of Materials and Methods. For the RNA structure dataset used in this work, the statistical results of base pairs including canonical and non-canonical ones are shown in Figure 4 and Supplementary Figure S4 in the Supplementary Material. For example, Figure 4B shows the geometric position (ρ,θ) distribution of the base U around its paired base A in A-U base pairs. Obviously, the base U appears frequently around base A at ρ∼7 Å and $\theta ∼0^{o}$ corresponding to the position of canonical Watson-Crick base pairs, while the other two high probability of occurrence positions are around $\theta ∼100^{o}$ and $\theta ∼280^{o}$ , where the two bases can interact through the Hoogsteen or sugar edge; see Supplementary Figure S3 in the Supplementary Material. Naturally, the base U is almost unobservable at $\theta \in \left(180,\;260^{o}\right)$ , where is occupied by the sugar. In contrast, the G-A base pair prefer to interact through the sugar edges; see Supplementary Figure S2 in the Supplementary Material. As shown in Figure 4D; Supplementary Figure S4 Supplementary Material, for the distribution of two stacking bases, e.g., adjacent C and G pairing with their complementary bases respectively, the base G occurs mainly above or below the base C with ρ∼3 Å, and $\theta ∼0^{o}$ (Butcher and Pyle, 2011; Bottaro et al., 2014). In addition, the 3D probability distribution for each base pair can also be present (Supplementary Figure S7 in the Supplementary Material), based on which, the 3D Gaussians for each possible Leontis-Westhof (LW) base pair type and for each applicable choice of two residue types can be fitted to obtain the corresponding mean and standard deviation; see Supplementary Table S6; Supplementary Figure S8 in the Supplementary Material.

Supplementary Figures S4–S8 in the Supplementary Material show the distributions for all the base-pairing and stacking, and the corresponding data files as well as fitting parameters ( ρ and θ for all base pairs with different LW types) can also be found at GitHub (https://github.com/RNA-folding-lab/RNAStat), which can be directly employed by the user to establish base-pairing/stacking potentials for RNA 3D structure prediction or evaluation.

#### 3.2.4 Distributions of the Distance Between Atoms

**FIGURE 7:**
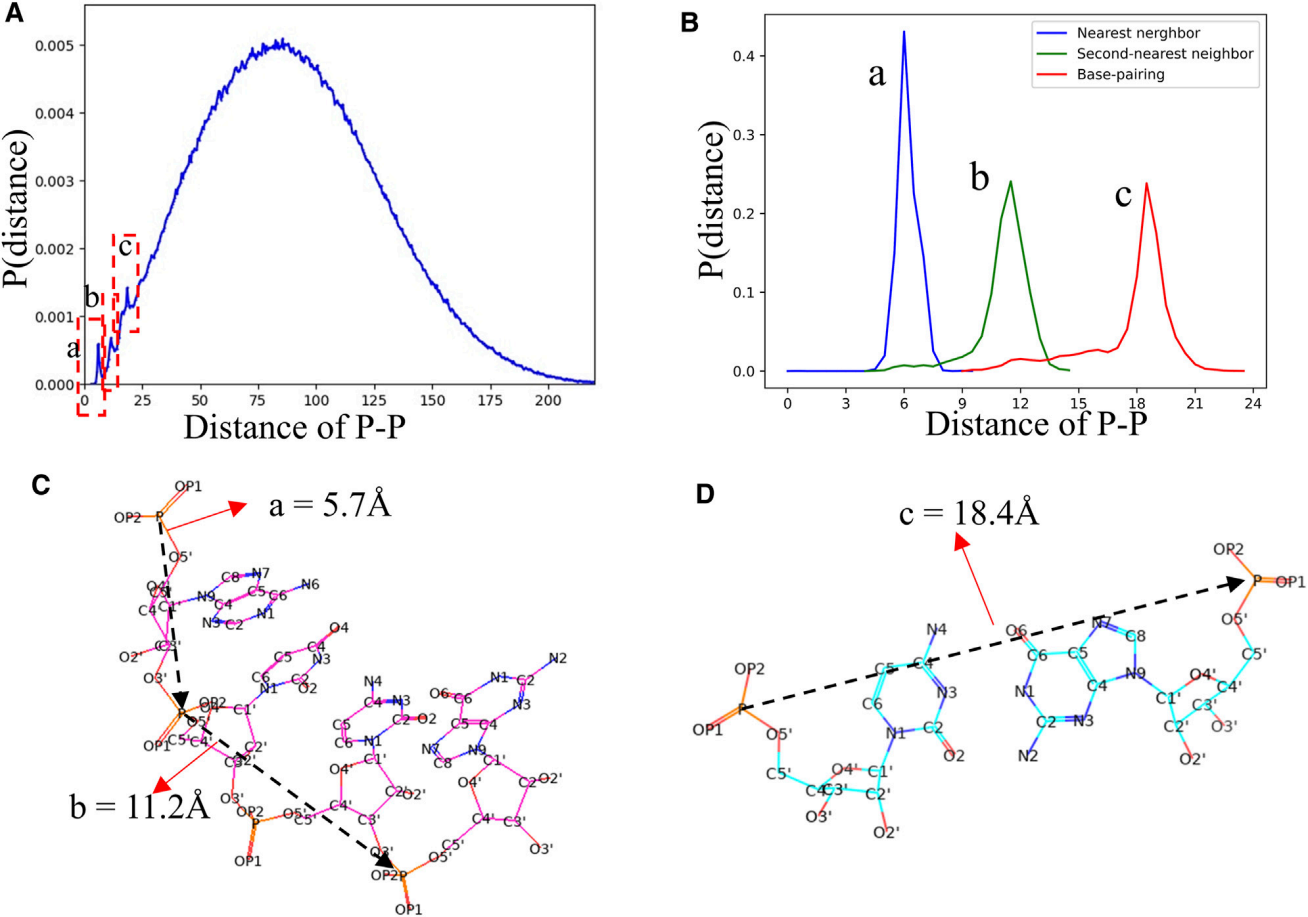
**(A)** The distance distribution between P atoms in our dataset. Three significant peaks are marked by dashed boxes. **(B)** The distance distributions between two P atoms in the nearest neighbor nucleotides (a, blue line), second-nearest neighbor nucleotides (c, green line), and paired nucleotides (c, red line), respectively. **(C, D)** Schematic diagram of the distances between P atoms in the nearest neighbor nucleotide, second-nearest neighbor nucleotides, and paired nucleotides. The a, b, and c in **(B–D)** are corresponding to the three peaks in **(A)**.

In view of the fact that most of the knowledge-based statistical potentials for RNA structure evaluation are based on the distances between atoms (Bernauer et al., 2011; Capriotti et al., 2011; Huang and Zou, 2011; Tan et al., 2019). The RNAStat can also be used to calculate the distance between any two non-bonded heavy atoms located at different nucleotides in RNA. For example, the distribution of distance between two atoms with type of P is shown in Figure 7A. In addition to a very broad peak at ∼70 Å, there are three noteworthy peaks at ∼5.7 Å, ∼11.2 Å, and ∼18.4 Å, respectively. The first two peaks are corresponding to the distances of two P atoms in the nearest neighbor nucleotides and next-nearest neighbor nucleotides, respectively, and the third peak represents distance between two P atoms in paired nucleotides; see Figures 7B,C. More distance distributions of atoms with various types can also be found in Supplementary Figure S9 in the Supplementary Material as well as data files at GitHub. Besides, the RNAStat tool also allows the users to input the atoms or atom types to perform statistical analysis for their distances; see in the section of Materials and Methods.

## 4 Conclusion

In summary, RNAStat is an integrated computational tool to perform comprehensive statistical analysis for the RNA 3D structures given by the users. The tool cannot only automatically calculate RNA global structural properties such as size and shape, but also analyze atom-atom distance distributions at atomic level. Furthermore, the tool can provide statistics of RNA secondary structure elements (e.g., canonical/non-canonical base pairs, stems and various loops) and geometric properties of base-pairing and base-stacking. In this work, we have established and utilized a non-redundant RNA 3D structure dataset to test the usability of the tool, and the statistical data could be directly used to build statistical potentials or energy functions for RNA 3D structure evaluation and prediction.

Still and all, further improvements need to be made on the tool to perform more detailed statistical analysis and to make it easier to use. For example, most of the available RNA statistical potentials generally adopt a distance-dependent scheme, however for proteins, the orientation-dependent statistical potentials, which consider the many-body interactions by statistically describing both distance and relative orientation between interacting atom groups, and have been proved to have better performance than the traditional distance-dependent potentials (Masso, 2018; Yu et al., 2019; Zhang et al., 2020). Thus, in the further development of RNAStat, the distribution of orientation (e.g., angle and torsion angle) between atoms as well as the joint probability at the given relative distance and orientation of observing two atoms should be taken into account. In addition, although the RNAStat is free-installation and convenient to use through command lines, it is still required the python installation or corresponding environment configuration. Thus, a user-friendly webserver could be further built after the deepened improvement for the tool. Very recent studies have shown that RNA scoring functions derived from deep learning of RNA 3D structures performed well in identification of accurate structural models (Kurgan and Zhou, 2011; Li et al., 2018; Wang et al., 2018; Huang et al., 2020; Townshend et al., 2021), which suggests that more potential structural features of RNAs should be further mined with the aid of deep neural networks.

## Data Availability

The original contributions presented in the study are included in the article/Supplementary Material, further inquiries can be directed to the corresponding author.
