# Supplementary material for: RNAStat: An Integrated Tool for Statistical Analysis of RNA 3D Structures
Source: Front Bioinform. 2022 Jan 11;1:809082. doi: 10.3389/fbinf.2021.809082 (PMC9580920; doi:10.3389/fbinf.2021.809082)
Supplement: Supplementary file 1 [file DataSheet1.PDF]

frontiers

Supplementary Material

1 Supplementary Figures and Tables

1.1 Supplementary Figures

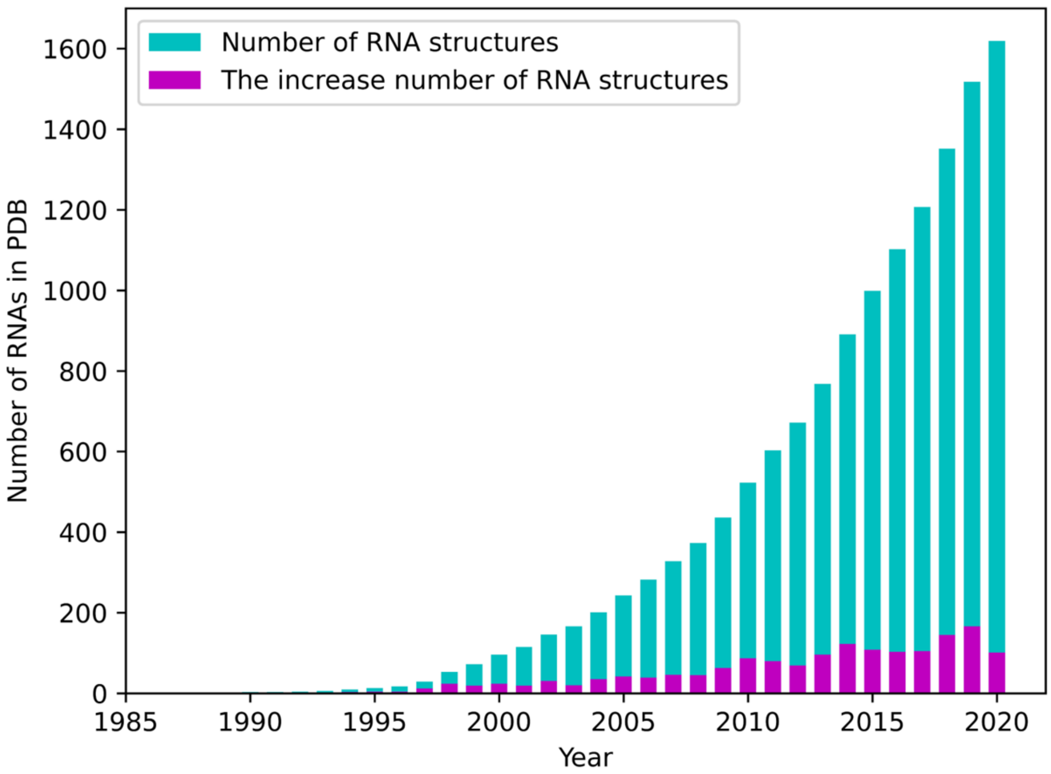

Figure S1. The evolution of the number of RNA structures in the PDB (https://www.rcsb.org/). The Figure is downloaded from the RNA 3D Hub set (Release nrlist_3.157_4.0Å). Green bar: the total number of RNA structures. Purple bar: the number of RNA structures released annually.

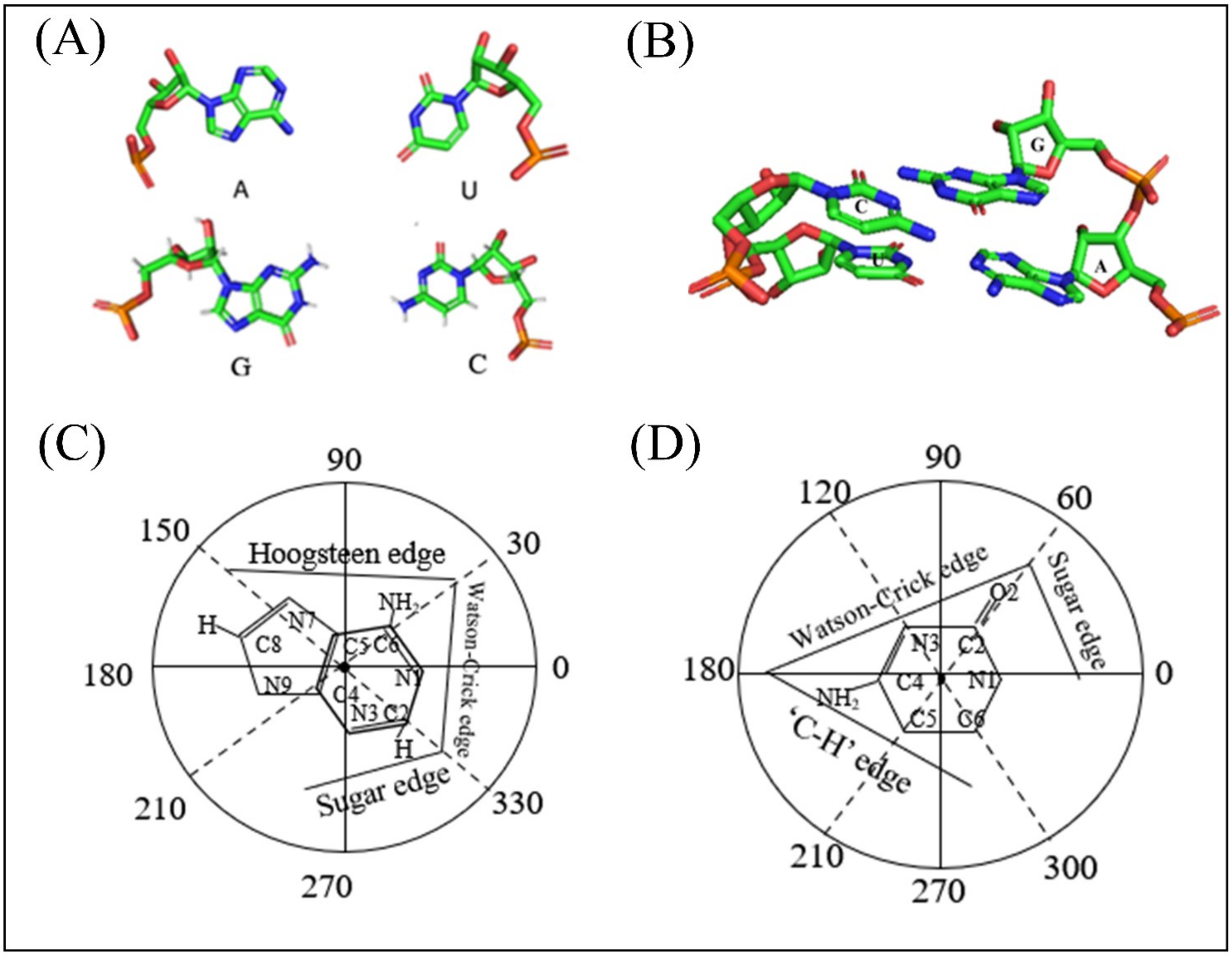

Figure S2. (A) The diagram of 3D structures for four types of nucleotides in RNA. (B) The diagram of geometry configuration of base-pairing and base-stacking. The 3D structures in (A) and (B) are shown with the PyMol (http://www.pymol.org) (C) Purine (A or G) bases provide three edges for interaction. As shown for adenosine, the Watson–Crick edge comprises N6, N1 and C2, with θ range from ~-60° to ~60 ^{o} in the coordinate system of the adenosine base; the Hoogsteen edge comprises N6 and N7; the Sugar-edge comprises C2 and N3. (D) Pyrimidine (C or U) bases provide three edges for interaction. As shown for cytosine, the Watson–Crick edge comprises O2, N3 and N4, with θ range from ~60° to ~180 ^{o} in the coordinate system of the cytosine base; the 'C-H' edge comprises N4 and C5; the Sugar-edge is around O2.

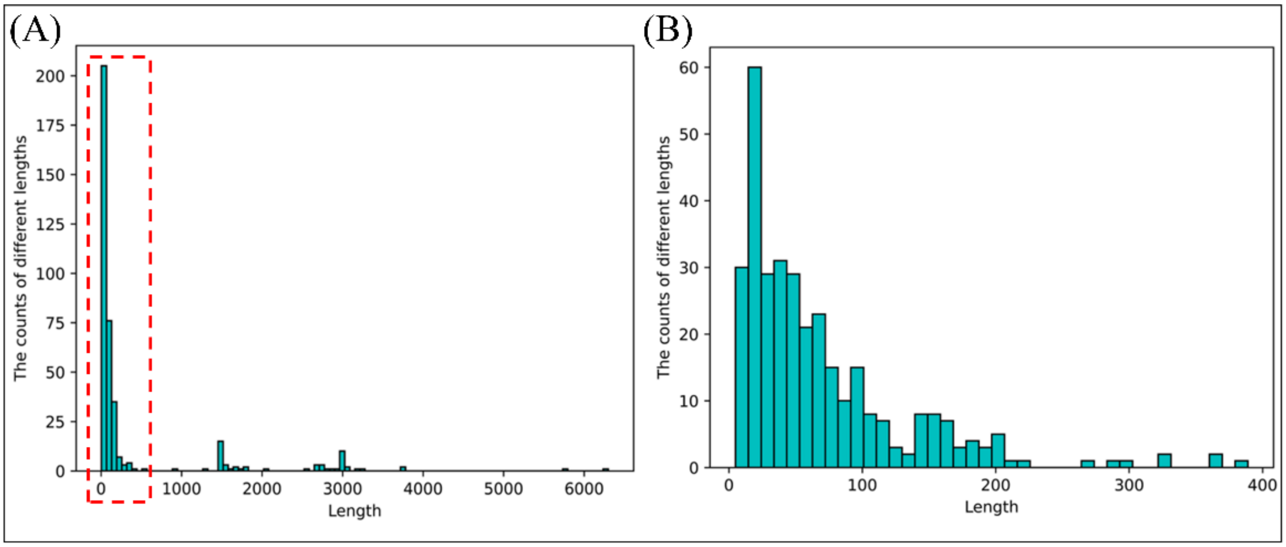

Figure S3. (A) Distribution of the length (*N*) of the 748 RNAs in the non-redundant dataset used in this work. The RNAs with length of *N*<400nt are marked with red dashed line, and the corresponding length distribution is shown in (B).

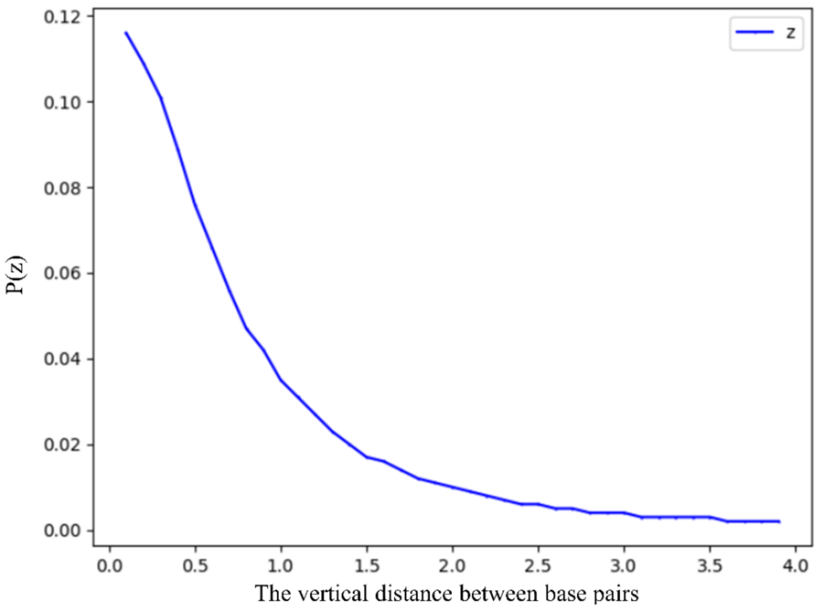

Figure S4. Distribution of |*z*| for base U in the coordinate system of its paired base A, that is, the distribution of perpendicular distance between two paired base planes.

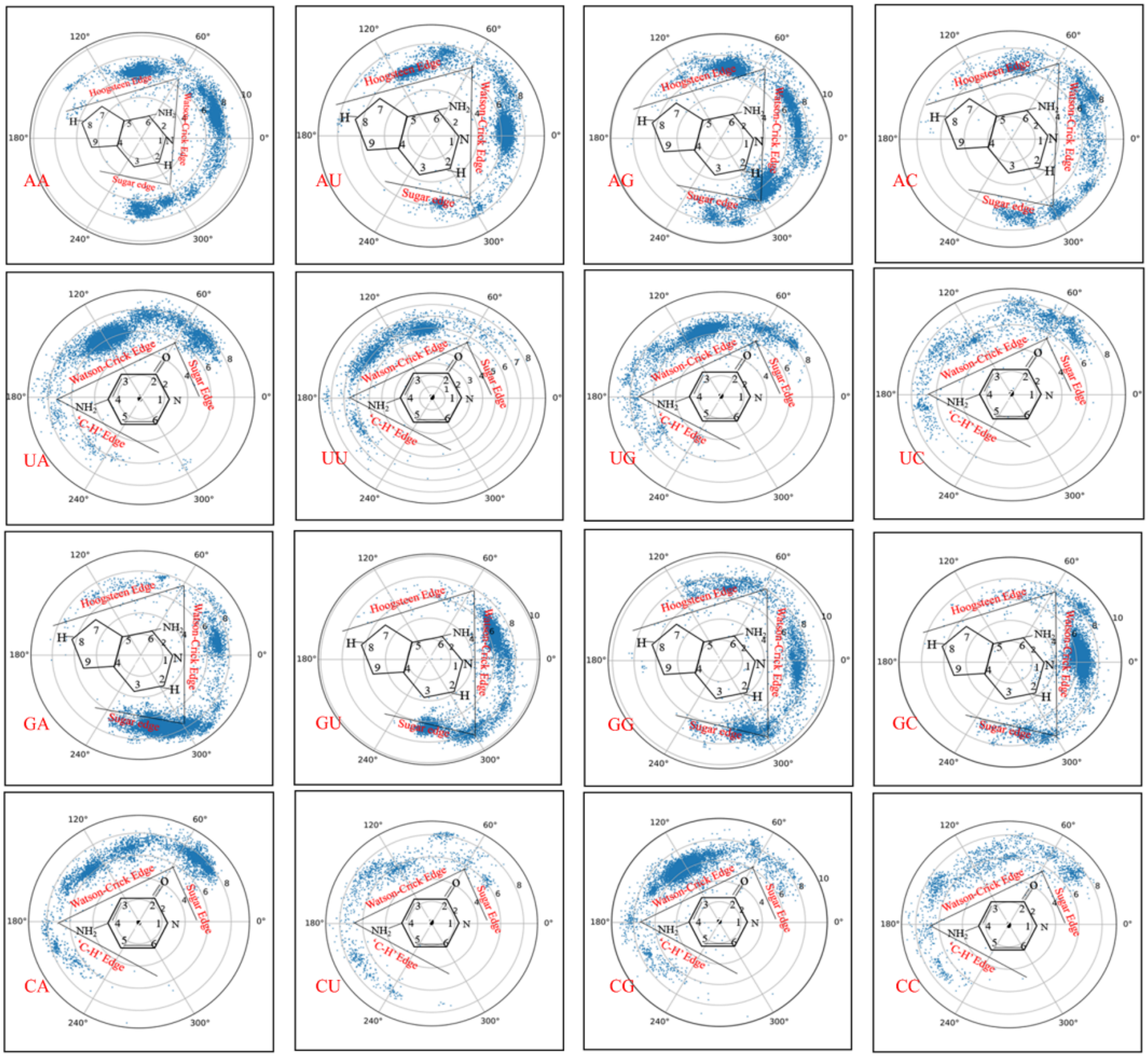

Figure S5. Distribution of (ρ, θ) for each base near its paired base (e.g., base-pairing between two adenosines in the first panel). All the 16 possible combinations between paired nucleotides are shown here.

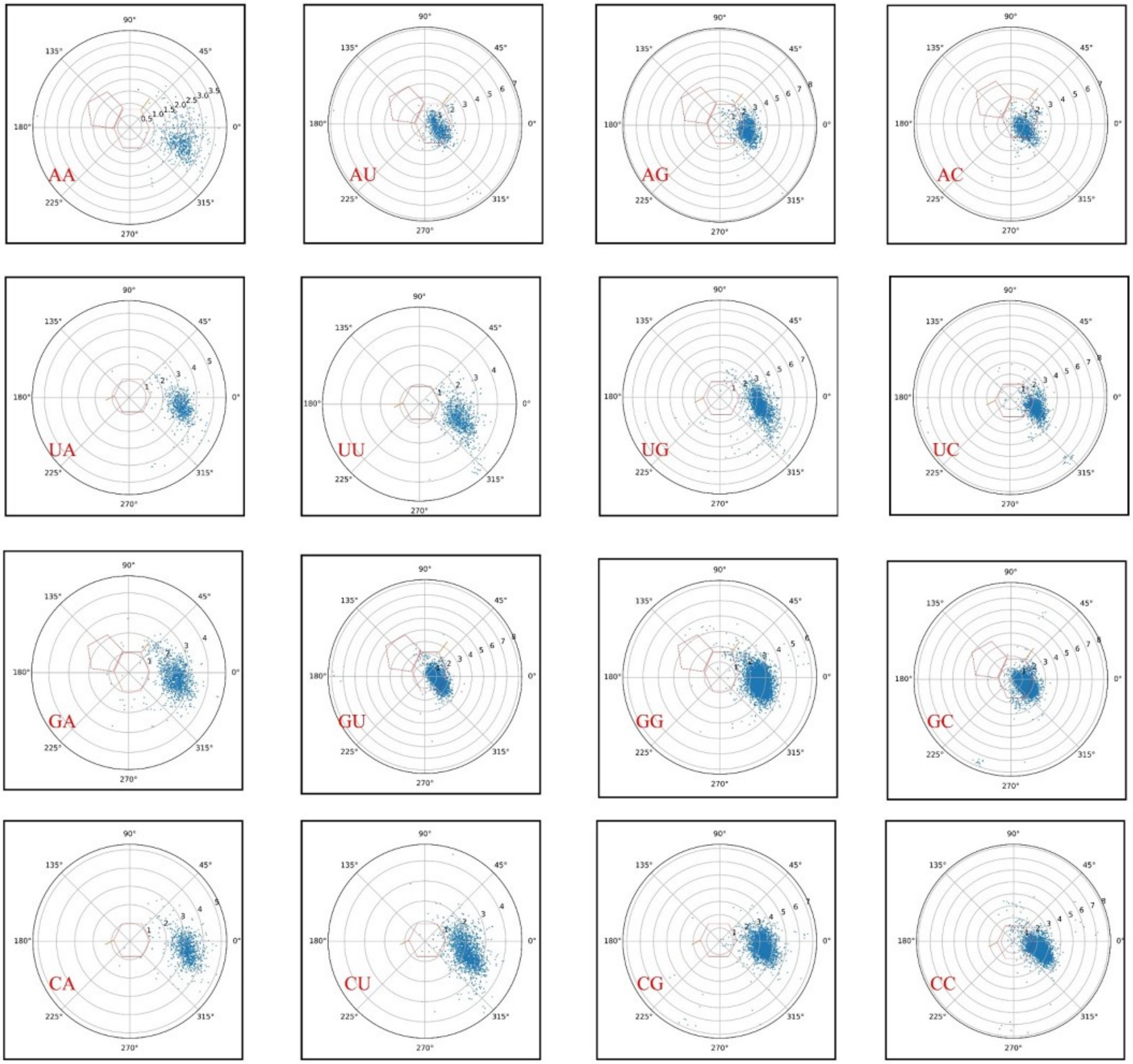

Figure S6. Distribution of (ρ, θ) for each base near its stacked base (e.g., base-stacking between two adenosines in the first panel). All the 16 possible combinations between nucleotides are shown here.

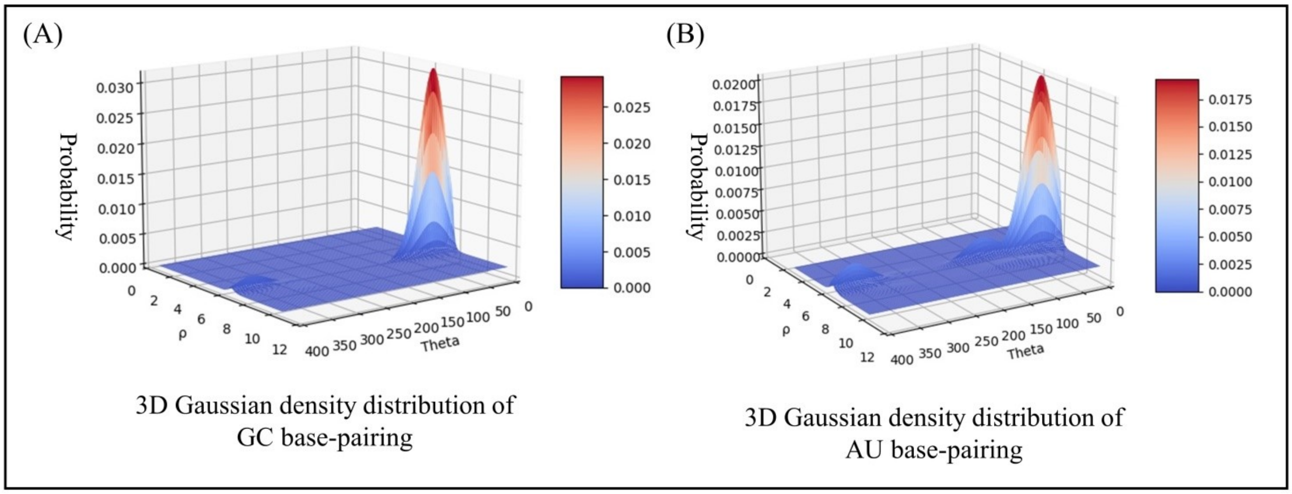

Figure S7. The 3D probability distributions for typical base pairs (A) G-C and (B) A-U.

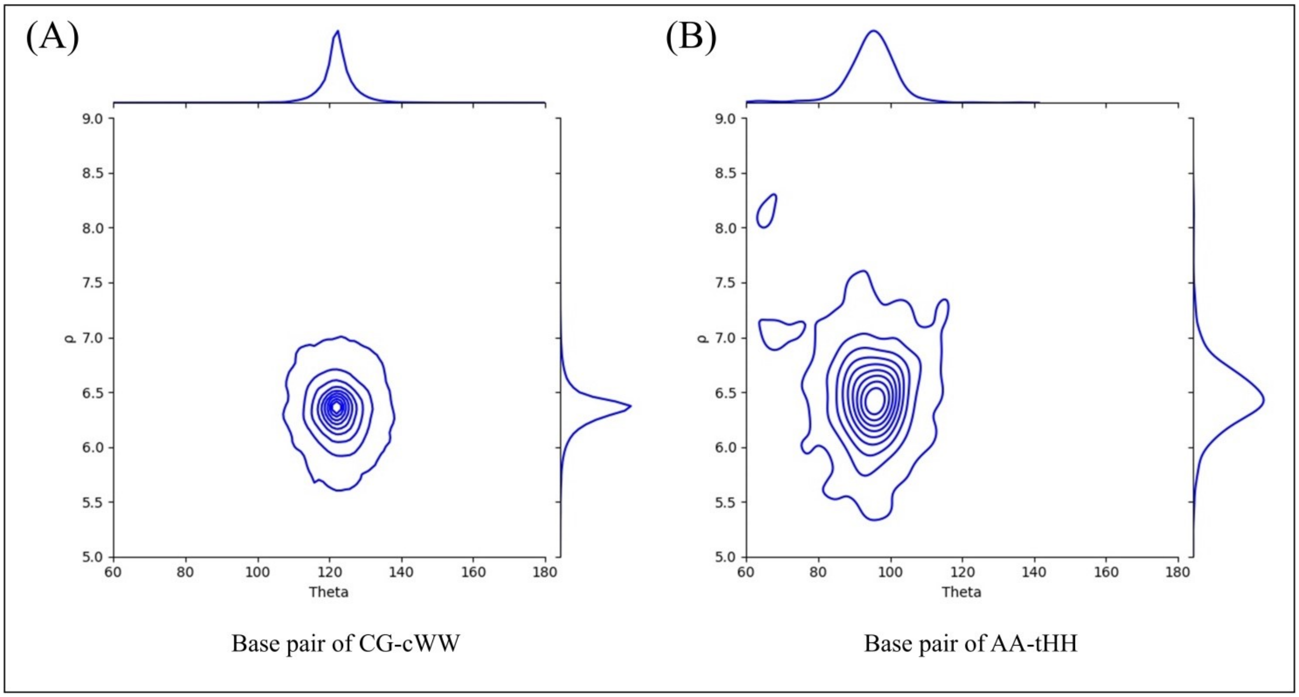

Figure S8. The schematic diagram of the Gaussian fitting for two example base pairs (A) C-G with cWW type and (B) A-A with tHH type.

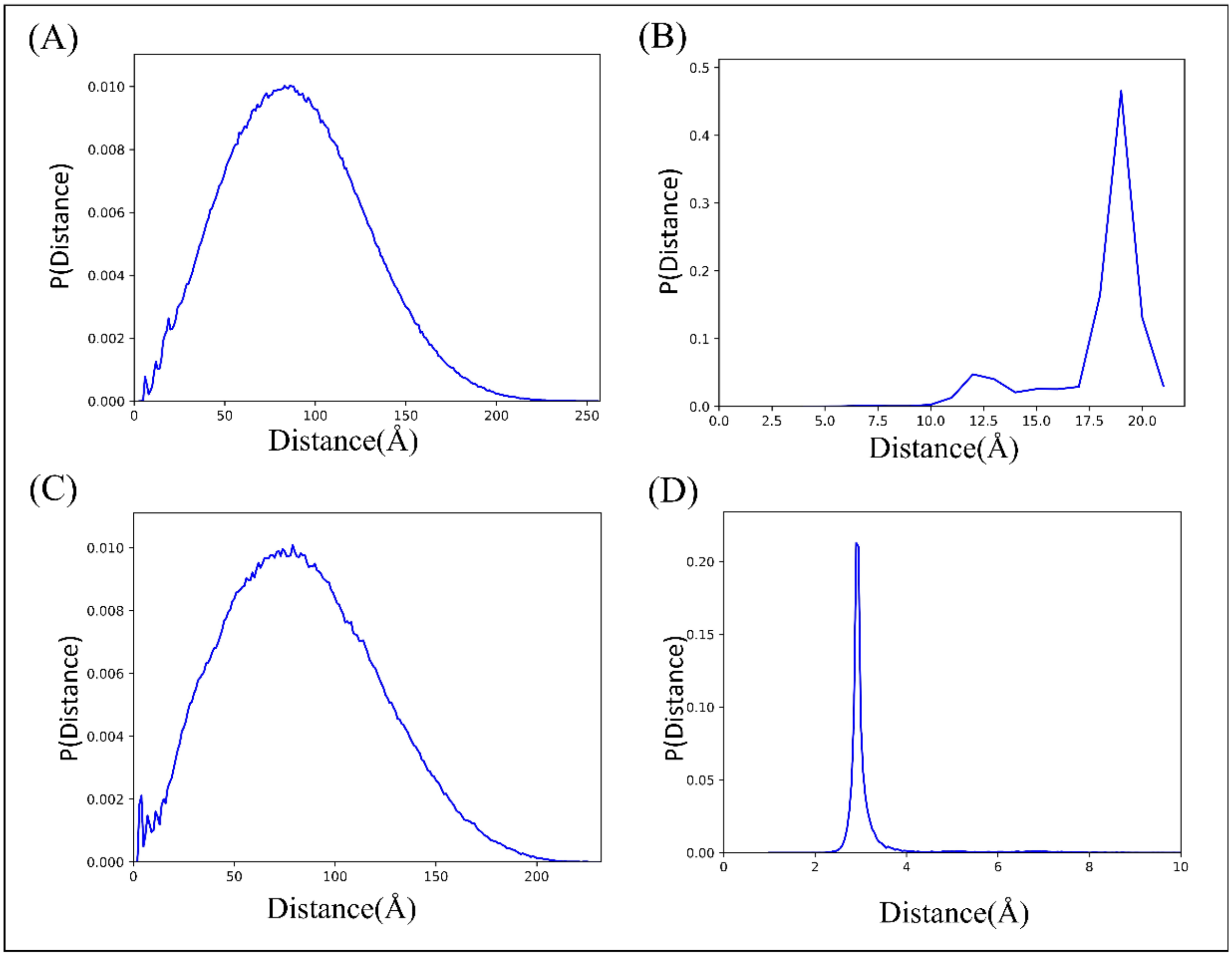

Figure S9. (A) The distance distribution between P atoms in nucleotides with bases of A and U, respectively. (B) The distance distribution between P atoms in A-U base pairs, and the two peaks are corresponding to that the two bases interact through the Hoogsteen and Watson-Crick edges, respectively. (C) The distance distribution between N3 atoms in cytosine and N1 atoms in guanine. (D) The distance distribution between N3 and N1 atoms in two paired nucleotides (C-G), corresponding to the hydrogen bond.

1.2 Supplementary Tables

Table S1. The PDB IDs of 783 RNAs in the non-redundant dataset established in this work.

1a9n	1yyw	2zi0	3og8	4ilm	4v99	5jc7	6aay	6mj0	6xur
1b23	1yz9	2zni	3oin	4ioa	4v9e	5jcf	6agb	6mkn	6xzd
1b7f	1yzd	2zue	3ovb	4j1g	4v9i	5jji	6ah3	6mpf	6ydp
1bmv	1z7f	2zy6	3oxe	4j39	4w5n	5js1	6ar1	6msf	6yft
1br3	1zbh	353d	3p59	4j50	4w90	5jup	6ar5	6mwn	6yl5
1c9s	1zbi	354d	3pdr	4jf2	4wan	5k36	6az3	6n2v	6ys3
1csl	1zci	359d	3pla	4jgn	4wfl	5k78	6bhj	6n4o	6ywe
1cvj	1zl3	361d	3po2	4jng	4wkr	5k7d	6bsg	6n5p	6yxy
1d4r	1zx7	364d	3ptx	4jrc	4wrt	5kk5	6c63	6n7r	6yym
1duq	1zz5	387d	3pu0	4jvh	4wsb	5kpy	6c6t	6nta	6yyt
1e8o	205d	397d	3pu1	4k0k	4wsm	5kvj	6cap	6nue	6z6m
1ec6	255d	3a6p	3q3z	4k27	4x4u	5l00	6cb3	6ny5	6z8k
1egk	280d	3add	3qjj	4k4u	4x62	5l2l	6ck5	6o1k	6zj3
1et4	283d	3aev	3r2c	4k50	4xjn	5lm7	6cu1	6o7h	6zmo
1f1t	2a43	3al0	3r4f	4kji	4xwf	5lta	6d12	6ol3	6zvk
1f27	2ann	3am1	3r9x	4knq	4y1m	5lys	6d3p	6ole	6zym
1f7u	2asb	3amu	3rw6	4kq0	4y4o	5lzs	6d6r	6oon	7aap
1f8v	2atw	3avu	3siv	4kre	4yaz	5m0i	6d8a	6ord	7b3c
1feu	2az0	3bnn	3sj2	4krf	4yye	5m3h	6d90	6ozo	7bzf
1fuf	2azx	3boy	3ski	4ktg	4z0c	5m73	6d9l	6p18	7c2k
1gtn	2b3j	3bsx	3sn2	4kxt	4z31	5ml7	6db9	6p2h	7c79
1h4s	2bh2	3cgs	3snp	4kyy	4z7k	5mrc	6dcl	6p5i	7cyq
1hmh	2bx2	3ciy	3ssf	4kzd	4z7l	5msf	6dlr	6pij	7d7v
1hys	2c0b	3cjz	3sux	4l8h	4zc7	5mwi	6dme	6pmo	7d81
1i6u	2c4q	3cul	3szx	4lck	4zdo	5ndi	6dn2	6qct	7jil
1i9x	2csx	3czw	3td0	4lmz	4zld	5nfv	6dnh	6qic	7jju
1il2	2czj	3d0m	3tup	4lvw	4zlr	5nwq	6dt8	6qn3	7kha
1j1u	2d6f	3d2v	3tzr	4m4o	4znp	5nxt	6dtd	6qw6	7msf
1j2b	2der	3dh3	3v6y	4m59	4zt0	5o5j	6du4	6qx9	
1jbr	2dlc	3dil	3v7e	4mcf	5amr	5o7h	6dvk	6r47	
1kd5	2ez6	3e5c	3vjr	4mgn	5aor	5ob3	6e1s	6r7g	
1kfo	2f8k	3egz	3w3s	4n0t	5aox	5ot2	6e7l	6r87	
1kog	2f8t	3eph	3wbm	4n2q	5awh	5t3k	6e8s	6rfl	

1kuq	2g92	3f2x	3wfs	4nfq	5axm	5t5a	6e9f	6rie
1kxk	2gic	3fs0	3x11	4ngf	5ay2	5t5h	6eri	6rip
1l2x	2gje	3ftm	3zc0	4nh3	5b2p	5t83	6f4g	6rja
1l9a	2gjw	3g9c	3zgz	4nha	5b63	5tbw	6f4h	6rr7
1lnt	2gtt	3gm7	3zla	4o26	5bjo	5tpy	6ff4	6rxu
1m5k	2h1m	3gs5	406d	4oji	5btp	5u30	6fpx	6s0x
1mhk	2hvy	3gtj	413d	4ol8	5bz5	5u3g	6fq3	6s8b
1mji	2hw8	3gtl	422d	4oo1	5bzu	5ud5	6fql	6sjd
1mzp	2i82	3hax	433d	4oog	5c0y	5udz	6fz0	6skg
1n32	2i91	3hhn	4a93	4oqu	5c45	5ued	6g3b	6sty
1nta	2il9	3hjf	4ang	4p95	5c5w	5uk4	6gaw	6svs
1nuv	2ix1	3hjw	4ato	4pcj	5c9h	5une	6gaz	6sx2
1ooa	2iy5	3ho1	4ay2	4pdb	5ccb	5uz6	6gc5	6sy6
1p6v	2izn	3hou	4b3o	4phy	5cki	5v3f	6gd2	6szu
1q2r	2j0s	3htx	4b3r	4pjo	5cnr	5vci	6gmh	6t0v
1q96	2jea	3iab	4bhh	4pkd	5czz	5vj9	6gsk	6t4q
1qbp	2jlt	3ibk	4boc	4pmi	5d0a	5vm9	6gsl	6t7t
1qc0	2jlv	3ice	4bw0	4pmw	5d8h	5voe	6h0r	6tff
1qcu	2nue	3iev	4bwm	4pqu	5dcv	5vpo	6ha8	6thn
1qtq	2nug	3igi	4bxx	4pqv	5ddo	5vzj	6hau	6tpq
1qvf	2o3x	3iwn	4by7	4pr6	5de8	5w0o	6hc5	6tqa
1r3e	2o5i	3j6b	4c4w	4prf	5dh6	5wlh	6hct	6tqb
1r9f	2oe8	3j79	4c7o	4py5	5dhc	5wnp	6hiw	6ty9
1rna	2oeu	3j7a	4c8y	4q0b	5dv7	5wnt	6htu	6tz2
1rpu	2oiu	3jbv	4cs1	4qcl	5e3h	5wti	6hu6	6u8d
1s03	2ozb	3jcs	4db2	4qi2	5e6m	5wty	6ia2	6u9x
1s0v	2pjp	3k0j	4e48	4qil	5ed1	5wwt	6ifl	6ufh
1sdr	2pn3	3k1v	4e5c	4qlm	5eeu	5wzj	6iqw	6ufj
1sds	2py9	3kfu	4ed5	4qqb	5ew4	5wzk	6iv9	6ugg
1ser	2q1o	3ks8	4enc	4qyz	5f5f	5x2g	6j9e	6uq3
1t0e	2qus	3loa	4erd	4r4v	5f5h	5x6b	6jdv	6uvn
1t0k	2qux	3lqx	4eya	4rbq	5f9f	5xc6	6jji	6v3a
1tfw	2qwy	3m3y	4f02	4rdx	5fj4	5xj2	6joo	6v4x
1tfy	2r1s	3mei	4f3t	4rge	5fmz	5xog	6jq5	6v9b
1u1y	2r8s	3mij	4frn	4rmo	5g4t	5xpg	6jvx	6vff
1u63	2rfk	3mja	4fts	4rum	5g4u	5xwg	6jxm	6vmi
1u6b	2uwm	3moj	4fyd	4rwn	5guh	5xwy	6kl9	6vqv

1u9s	2uxc	3mqk	4gg4	4rzd	5h1k	5xy3	6kuj	6vrc
1un6	2vum	3ncu	4gha	4tue	5hby	5y58	6kyv	6vwl
1wz2	2w2h	3nd3	4ghl	4tuw	5hjz	5y7m	6l5n	6w2t
1xjr	2xb2	3ndb	4gxy	4u7u	5hk0	5y88	6lax	6w5c
1xnq	2xdb	3nkb	4h5p	4uyk	5hsw	5z1i	6lse	6w6p
1xnr	2xlk	3nmr	4h8k	4v2s	5i4a	5zeb	6ltu	6wbr
1xok	2xnr	3nnh	4hkq	4v50	5i9d	5zkj	6m0x	6woo
1y27	2y8w	3npq	4ifd	4v5k	5j7l	5ztm	6m62	6wyb
1yfg	2ygh	3nvk	4ig8	4v83	5jaj	5zw4	6mcb	6x5m
1yls	2z75	3o7v	4ill	4v8q	5jbg	5zwm	6mfn	6xh2

Table S2. 85 heavy atom types in four types of nucleotide (A, U, G, C).

**A**	P, OP1, OP2, O5', C5', C4', O4', C3', O3', C2', O2', C1', N9, C8, N7, C5, C6, N6, N1, C2, N3, C4
**U**	P, OP1, OP2, O5', C5', C4', O4', C3', O3', C2', O2', C1', N1, C2, O2, N3, C4, O4, C5, C6
**G**	P, OP1, OP2, O5', C5', C4', O4', C3', O3', C2', O2', C1', N9, C8, N7, C5, C6, O6, N1, C2, N2, N3, C4
**C**	P, OP1, OP2, O5', C5', C4', O4', C3', O3', C2', O2', C1', N1, C2, O2, N3, C4, N4, C5, C6

Table S3. The probability of the occurrence of different nucleotides (A, U, G, and C) in our dataset.

	**A**	**U**	**G**	**C**	**Total**
**F** [a]	0.23	0.18	0.34	0.25	1.0
**NUM** [b]	79828	61389	120220	86192	348476

[a] The frequency of the occurrence of each base.

[b] The number of the occurrence of each base.

Table S4. The probability of the occurrence of all possible base pairs including canonical and non-canonical ones.

	A	U	G	C
**A**	0.038 [a]	0.092	0.059	0.019
	6586 [b]	16024	10195	3368
**U**	0.115	0.017	0.044	0.011
	19977	2908	7590	1882
**G**	0.071	0.050	0.026	0.211
	12417	8791	4465	36788
**C**	0.023	0.001	0.203	0.010
	4000	1281	35413	1710

[a] The frequency of the occurrence of the base pair.

[b] The number of the occurrence of the base pair.

Table S5. The probability of the occurrence of all possible base-pair stacks.

	AU	UA	CG	GC	GU	UG
**AU**	0.021 [a]	0.023	0.034	0.038	0.004	0.007
	1595 [b]	1697	2551	2883	311	502
**UA**	0.025	0.021	0.037	0.041	0.006	0.005
	1857	1585	2745	3094	436	362
**CG**	0.034	0.038	0.129	0.080	0.008	0.018
	2561	2551	7130	9315	1132	2129
**GC**	0.034	0.034	0.095	0.124	0.015	0.028
	2561	2551	7130	9315	1132	2129
**GU**	0.006	0.007	0.024	0.022	0.003	0.001
	421	516	1765	1613	205	60
**UG**	0.004	0.004	0.015	0.010	0.005	0.002
	272	291	1142	734	382	114
**Total**	74925					

[a] The frequency of the occurrence of the base stack.

[b] The number of the occurrence of the base stack.

Table S6. The mean and standard deviation of ρ and θ for each possible Leontis-Westhof base pair type and for each applicable choice of two residue types.

	**cWW**	**tWW**	**cWH**	**tWH**	**cWS**	**tWS**
**AA**	7.26±0.53 [a]<br>4.99±19.85 [b]	7.45±0.39<br>18.83±11.21	7.13±0.51<br>356.34±22.59	7.01±0.39<br>12.58±12.55	7.20±0.42<br>18.87±34.26	6.72±0.48<br>12.82±40.68
**AU**	6.36±0.27<br>3.89±6.18	6.32±0.42<br>5.01±10.93	- [c]	-	7.54±0.55<br>357.9±38.40	7.53±0.35<br>342.5±25.89
**AG**	7.10±0.39<br>6.89±11.25	7.68±0.88<br>350.94±13.29	7.31±0.60<br>22.45±17.21	-	6.61±0.89<br>341.3±31.56	6.96±0.49<br>359.4±34.03
**AC**	6.89±0.68<br>17.78± 22.48	6.79±0.53<br>17.21±23.29	7.16±0.42<br>346.87±6.56	7.20±0.48<br>8.44±13.62	7.58±0.54<br>4.87±37.56	7.43±0.67<br>343.8±28.53
**UA**	6.34±0.29<br>120.45±7.60	6.33±0.56<br>113.58±14.49	6.03±0.60<br>118.93±13.95	6.10±0.56<br>109.5±12.10	5.92±0.36<br>117.6±12.10	5.94±0.30<br>118.6±15.20
**UU**	6.09±0.44<br>121.70±25.22	6.08±0.48<br>104.53±20.80	6.04±0.63<br>101.59±17.32	6.39±0.52<br>114.5±27.21	6.55±0.61<br>111.70±15.26	-
**UG**	6.53±0.36<br>103.95±12.41	6.79±0.58<br>12.07±30.85	5.97±0.78<br>110.71±18.01	6.43±1.24<br>108.1±28.56	7.03±0.93<br>132.2±32.98	6.59±1.05<br>111.89±30.52
**UC**	6.40±0.87<br>117.95±27.0	-	6.93±0.76<br>78.23±24.13	7.09±0.66<br>80.37±17.74	-	-
**GA**	7.04±0.49<br>9.56±10.80	7.54±0.83<br>340.15±21.58	7.05±0.86<br>12.37±19.02	8.04±0.85<br>325.7±24.12	6.82±0.79<br>342.5±30.21	7.25±0.94<br>334.2±25.80
**GU**	6.55±0.40<br>21.78±11.35	7.60±0.95<br>345.05±31.97	7.60±1.01<br>132.88±19.81	7.45±0.78<br>337.4±26.57	-	7.53±0.61<br>351.5±24.76
**GG**	7.26±0.76<br>5.62±20.20	7.26±0.80<br>10.24±21.66	7.17±0.53<br>350.00±10.09	7.20±0.64<br>349.9±22.42	7.69±0.77<br>19.96±25.06	-
**GC**	6.37±0.25<br>5.35±6.03	6.76±0.56<br>350.91±14.74	7.02±0.95<br>355.44±26.01	-	-	7.63±0.63<br>338.6±22.71
**CA**	6.68±0.66<br>105.24±20.45	6.58±0.53<br>120.54±19.00	6.83±0.61<br>98.95±17.38	6.47±0.53<br>127.6±19.25	6.87±0.49<br>152.21±9.30	-
**CU**	6.23±0.82<br>126.71±18.93	6.35±0.93<br>96.04±31.00	-	7.59±0.80<br>90.10±18.64	7.10±0.61<br>147.1±12.99	-
**CG**	6.33±0.26<br>122.21±6.14	6.55±0.55<br>99.48±13.59	6.16±0.91<br>120.15±22.75	6.75±0.50<br>137.3±23.66	6.75±0.74<br>139.0±29.83	7.46±0.89<br>102.7±40.83
**CC**	6.32±0.74<br>116.32±25.95	6.54±0.82<br>134.34±24.31	6.38±0.89<br>85.21±15.50	6.48±1.04<br>94.51±17.34	7.14±0.59<br>149.26±9.27	-

(Continued table)

	cHH	tHH	cHS	tHS	cSS	tSS
**AA**	7.57±1.02<br>123.2±30.17	6.46±0.38<br>94.59 ±7.74	6.77±0.58<br>72.68±7.91	6.59±0.51<br>86.94±15.27	7.11±0.33<br>285.3±20.51	-
**AU**	6.53±0.86<br>96.25±29.20	7.52±0.61<br>86.99±13.73	7.27±0.40<br>85.05±12.97	7.27±0.40<br>0.96±26.79	7.44±0.45<br>293.5±35.59	-
**AG**	7.07±0.79<br>87.66±22.06	7.35±0.47<br>83.82±12.98	6.93±0.41<br>82.55±30.56	6.89±0.31<br>82.41±8.78	7.44±0.81<br>272.3212.97	6.11±0.73<br>303.5511.09
**AC**	-	7.15±0.70<br>93.62±9.78	6.44±0.45<br>89.82±29.21	7.15±0.80<br>89.17±22.33	7.32±0.43<br>297.11±8.34	-
**UA**	5.95±0.95<br>217.1±17.10	6.66±0.67<br>200.32±36.30	-	-	7.32±0.43<br>63.07±6.33	-
**UU**	-	-	-	-	-	-
**UG**	-	-	-	-	7.90±0.57<br>40.40±7.71	-
**UC**	6.90±0.66<br>206.50±5.85	-	-	-	-	-
**GA**	6.83±0.90<br>123.6±28.76	7.27±0.71<br>89.08±31.41	-	-	7.85±0.44<br>301.1±12.15	6.60±0.44<br>280.22±3.96
**GU**	5.95±1.04<br>77.18±14.36	-	-	-	7.96±0.47<br>295.31±4.95	-
**GG**	7.22±0.58<br>85.60±20.27	-	7.20±1.06<br>89.80±20.67	7.63±0.96<br>82.04±20.58	7.19±0.65<br>270.9±21.35	6.88±0.56<br>288.62±6.44
**GC**	6.31±1.39<br>111.9±33.05	-	-	-	7.62±0.54<br>289.10±8.76	-
**CA**	-	6.41±0.50<br>217.12±13.15	-	-	7.52±0.46<br>38.03±3.84	-
**CU**	6.77±0.25<br>11.32±7.73	-	-	-	-	-
**CG**	6.43±0.91<br>215.6±15.81	7.06±0.49<br>208.45±8.54	-	-	7.58±0.80<br>36.23±1096	-
**CC**	-	-	-	7.05±0.39<br>211.87±8.82	-	-

[a] The mean ± standard deviation of ρ.

[b] The mean ± standard deviation of θ.

[c] The number of corresponding base pairs is less than 50.
